# Influence of Superimposition Strategies and Landmark Distribution on the Full‐Arch Trueness of Orthodontic Digital Models

**DOI:** 10.1111/jerd.70179

**Published:** 2026-05-07

**Authors:** Ezgi Cansu Firinciogullari, Edanur Dark, Genta Agani Sabah, Aslıhan Mediha Erdinc

**Affiliations:** ^1^ Department of Applied Craniofacial Sciences Medical University of South Carolina Charleston South Carolina USA; ^2^ Private Practice Izmir Türkiye; ^3^ Department of Orthodontics, Faculty of Dentistry Izmir Tinaztepe University Izmir Türkiye; ^4^ Department of Orthodontics School of Dentistry, Ege University Izmir Türkiye

**Keywords:** 3D, best‐fit, desktop scanner, intraoral scanner, precision, superimposition, three‐point alignment, trueness

## Abstract

**Objective:**

To evaluate the influence of superimposition protocols and landmark distribution on deviation outcomes in orthodontic full‐arch models.

**Materials and Methods:**

Twenty plaster models were scanned using an intraoral and desktop scanner. Ten models were evaluated for landmark suitability and inter‐scanner coordinate agreement. All models were superimposed using automatic global best‐fit (AGB), three‐point landmark (3PL) alignment with two different triads, and area‐specific alignment (ASA). Deviation metrics were root mean square (RMS), absolute average (Abs. Avg.), and (90–10)/2. Mixed‐effects models were utilized for analysis.

**Results:**

The alignment protocol significantly affected all metrics (*p* < 0.001). The protocol × arch interaction was significant for RMS (*p* < 0.001) and (90–10)/2 (*p* = 0.046), but not Abs. Avg. (*p* = 0.484). AGB and both 3PL triads produced comparable outcomes (*p* > 0.05), whereas ASA yielded higher deviations (*p* < 0.01), particularly in the mandible (RMS increased by approximately 27 μm relative to AGB).

**Conclusions:**

AGB and 3PL protocols yield comparable reference‐based trueness, whereas ASA produced higher deviations. This effect is strongly arch‐dependent, suggesting the unreliability of restricted‐region registration in mandibular datasets.

**Clinical Significance:**

Digital superimposition trueness depends heavily on the alignment protocol. Standardizing registration is essential, as minor positional differences impact symmetry and finishing decisions.

## 1 Introduction

Three‐dimensional (3D) digital superimposition has become a cornerstone of contemporary dental research, enabling objective quantification of treatment‐related changes and verification of manufacturing fidelity. In the dental field, 3D superimposition is routinely used to monitor tooth movement and arch form changes, evaluate aligner and prosthesis adaptation, assess the relative position of implants, and, in craniofacial applications, compare pre‐ and post‐intervention morphology in growth‐ or surgery‐related assessments [1, 2, 3]. Yet, despite its wide adoption, 3D superimposition is not a single standardized procedure, with the numerical outputs it produces inherently shaped by how two surfaces are brought into correspondence [4].

Under ISO 5725 terminology, accuracy comprises trueness and precision, two complementary properties that are not solely scanner‐dependent but are also influenced by the reference dataset selected and the superimposition strategy used to establish surface correspondence [5]. In practice, 3D model superimposition is commonly performed using one of three conceptual approaches. Automated surface‐based alignment iteratively converges on an optimal fit based on shared geometry. Landmark‐guided registration uses user‐identified anatomical points to initialize the relative pose of the datasets. Conversely, region‐restricted or area‐based registration constrains the optimization to a user‐defined anatomical zone. Importantly, these options are not merely technical preferences, as changing the registration paradigm alone can shift the resulting deviation metrics [4, 6], with prior studies showing that discrepancies become more pronounced when registration relies on reduced or unilateral reference regions or in morphologically constrained/partially dentate scenarios [6, 7, 8]. Findings from a controlled defect‐model study suggest that although global best‐fit is widely adopted, its optimization logic can bias deviation metrics when true change is present [9]. Meanwhile, landmark‐based workflows retain intuitive appeal but remain susceptible to operator subjectivity [10] and are frequently implemented as hybrid procedures in which landmark initialization is followed by automated surface refinement. Nevertheless, there is no consensus on a universally reliable set of dental landmarks, and systematic evidence indicates that no landmark‐based method has yet been shown to consistently deliver reliable results [4, 11, 12, 13]. Finally, much of the existing evidence base derives from idealized typodonts, semi‐arch models, or prosthodontic simulations, which may not generalize to the morphological variability encountered in clinically derived full‐arch datasets [4, 6, 9, 10]. As landmark guidance is used for initial positioning and is subsequently followed by automated surface refinement, making deviation outcomes sensitive to both the initialization and the optimizer's convergence behavior [6, 7, 9, 14]. Moreover, registration behavior may be axis‐dependent, with discrepancies reported to be more pronounced in the apicocoronal dimension under certain processing conditions [15].

Based on existing evidence from partial‐arch and typodont studies, it can be anticipated that restricting alignment to specific anatomical areas may yield deviation metrics different from those of global best‐fit or landmark‐guided methods [2, 8, 16, 17, 18, 19]. Currently, there is a lack of data on how distinct superimposition paradigms behave in clinically derived full‐arch models, which naturally exhibit minor asymmetries, rotational discrepancies, and variations in arch form. Furthermore, because the maxilla contains relatively stable anatomical structures such as the palatal rugae, whereas the mandible lacks a universally reliable reference region, the literature suggests that alignment behavior may be highly arch‐dependent [20, 21, 22]. Therefore, the present in vitro study was designed to: (1) validate candidate control landmarks by quantifying landmark repeatability and post‐registration inter‐scanner coordinate agreement (Phase I); (2) quantify reference‐based trueness and test the impact of four different superimposition protocols on deviation outcomes, specifically referring to two alternative three‐point landmark (3PL) configurations within a refinement‐based workflow (Phase II), and (3) evaluate maxillary and mandibular alignment behavior separately. Building on the existing scientific evidence, the research hypotheses were that: (1) full‐arch deviation outcomes would differ according to the superimposition protocol, with area‐specific alignment (ASA) expected to produce greater deviations than automatic global best‐fit (AGB) and 3PL‐based registration; and (2) deviation outcomes would differ between the maxillary and mandibular arches, and the effect of the superimposition protocol would vary by arch.

## 2 Materials and Methods

**FIGURE 1:**
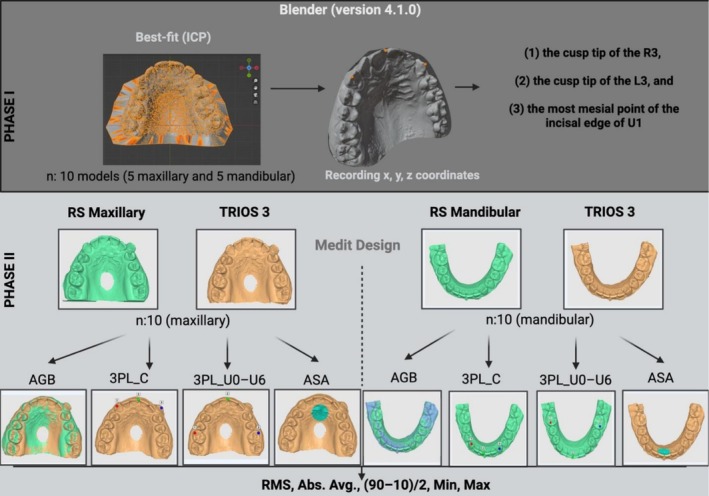
Workflow of the study.

This in vitro investigation was conducted in two sequential phases. Phase I was designed to (i) screen and validate candidate dental landmarks for use as control points in the 3PL alignment and (ii) quantify post‐registration inter‐scanner coordinate agreement at these landmarks, based on morphological distinctiveness, coordinate stability after rigid registration, and intra‐observer repeatability. Phase II evaluated trueness and quantified alignment‐dependent variation in deviation outcomes across three distinct superimposition paradigms. Ethical approval for this study was obtained from the Institutional Review Board of Ege University (Approval No. 25‐6T/76). All patient‐derived records were fully anonymized prior to inclusion in the study. A total of 30 models (15 maxillary and 15 mandibular) were included in the study (Figure 1).

Plaster models were selected from orthodontic records of patients who had presented for treatment. Only patients with permanent dentition and no missing teeth were included. To minimize morphological extremes that could artificially affect surface matching, total crowding was limited to < 6 mm. Cases with diastema, severe overlapping, or complex interproximal stacking were excluded. Consequently, crowding within the included sample was primarily characterized by mild deviations from the ideal arch perimeter.

Each model was scanned using both an intraoral scanner (IOS) (TRIOS 3; 3Shape, Copenhagen, Denmark) and an extraoral desktop scanner (EOS) (DuraScan Alpha; ERK Dental/Erk Digital, İzmir, Türkiye). The TRIOS 3 device was calibrated according to the manufacturer's instructions before scanning sessions, and all scans were performed by a single experienced operator (E.D.) following a standardized scan path that began distally on the molar occlusally, progressed across the arch, and subsequently captured the lingual/palatal and buccal surfaces.

The DuraScan Alpha operates via phase‐shifting optical triangulation and provides a stated point resolution of 10 μm in accordance with ISO 12836‐related specifications. The device was calibrated using the manufacturer‐provided calibration object before acquisition. To assess scanner repeatability, five models were scanned three times with both the DuraScan Alpha and TRIOS 3 before the main study. These repeated scans were used to estimate within‐scanner precision and to support subsequent trueness analyses. Precision was quantified by performing all pairwise STL–STL comparisons among the three scans (Scan1–Scan2, Scan2–Scan3, and Scan1–Scan3). Deviation metrics obtained from these pairwise superimpositions were used to summarize repeatability at both the model and scanner levels.

### 2.1 Phase I: Pilot Study, Inter‐Scanner Agreement, and Repeatability

A pilot analysis was conducted on a subset of 10 models (5 maxillary and 5 mandibular) to screen candidate landmarks prior to their use as control points in the 3PL protocol. Candidate landmarks were selected a priori based on clinical identifiability and morphological distinctiveness. For pilot screening and coordinate‐based agreement assessment, paired STL datasets (TRIOS 3‐IOS vs. DuraScan Alpha‐EOS) were imported into Blender (version 4.1.0; Blender Foundation, Amsterdam, The Netherlands) and registered before coordinate extraction. To enable consistent coordinate capture, each pair of datasets first underwent rigid pose normalization using the iterative closest point (ICP) algorithm to account for arbitrary differences in initial orientation and position. This procedure placed the paired models into a common Cartesian coordinate system, after which the three‐dimensional coordinates (*x*, *y*, and *z*) of three predefined landmarks were recorded for each scanner dataset: (1) cusp tip of the right canine, (2) cusp tip of the left canine, and (3) the most mesial point of the incisal edge of the right central incisor were recorded for each scanner dataset. Inter‐scanner landmark agreement was quantified using three‐dimensional Euclidean deviation (Δ3D) for each landmark, and paired coordinate differences between scanners were assessed. Landmark identification was repeated by the same investigator (E.D.) after a 2‐week interval under identical conditions (Figure 1). Inter‐scanner agreement in Phase I refers to coordinate‐level consistency after registration, rather than absolute accuracy of either scanner. Phase I was intended as a methodological validation step to assess landmark suitability and repeatability and was not designed to evaluate scanner accuracy or the performance of the alignment protocol.

### 2.2 Phase II: Accuracy of Superimposition Protocols

STL files of 20 full‐arch models (10 maxillary and 10 mandibular), distinct from those used in Phase I, were imported into Medit Design (Medit, Seoul, Korea). Before superimposition, digital models were trimmed approximately 5 mm apical to the gingival margin using consistent boundaries across all datasets. For the main analysis, DuraScan Alpha datasets served as the reference standard (reference dataset), and TRIOS 3 datasets were treated as test scans for trueness estimation.

Four alignment protocols were evaluated: (1) AGB, fully automated global surface alignment using the software's built‐in best‐fit algorithm; (2) 3PL alignment using the pilot‐validated landmark triad (3PL_C); (3) 3PL alignment using the widely [23, 24] adopted U0–U6 reference triangle [25] (3PL_U0–U6; U0: incisal midpoint between the central incisors; U6: mesiobuccal cusp tips of the right and left first molars); (4) ASA, anatomically restricted surface alignment. For maxillary models, the second medial palatal rugae region was selected as the reference area, whereas for mandibular models, a restricted region including the lingual‐incisal surface of the central incisors and adjacent gingiva was used from the lingual view (Figure 1).

Following alignment, surface deviations were computed using standardized thresholds (±100 μm; tolerance 10 μm). No additional manual adjustments to the software alignment settings were made during the analysis. User input was limited to the selection of the predefined landmark triads and the anatomically restricted reference areas for the corresponding protocols, whereas the subsequent alignment and deviation analysis were carried out automatically by the software. The standardized color‐map thresholds were used for visualization only and did not influence the quantitative deviation calculations. Quantitative deviation metrics included root mean square deviation (RMS), absolute average deviation (Abs. Avg.), and the (90–10)/2 percentile half‐range. Intra‐observer repeatability was evaluated by reevaluating all registration protocols on 40% of the sample (*n* = 8) after 14 days by the same researcher.

### 2.3 Statistical Analysis

An a priori sample size calculation was performed using G*Power software (version 3.1.9.7, G*Power; Kiel University, Kiel, Germany). Because G*Power does not natively support power estimation for mixed‐effects models, a repeated measures analysis of variance (ANOVA) with a within‐between interaction was utilized as a proxy. The calculation was based on an assumption of two groups (maxillary and mandibular arches) and four repeated measurements (the four alignment protocols), with an estimated correlation among repeated measures of 0.70 and a nonsphericity correction (*ε*) of 1. Assuming a medium effect size (*f* = 0.25), a 0.05 significance level, and a target power of 0.80, the minimum required total sample size was determined to be 16 models. Therefore, the inclusion of 20 full‐arch models (10 maxillary and 10 mandibular) was deemed sufficient to provide adequate statistical power to detect significant protocol‐by‐arch interactions and main effects for the primary outcomes.

Normality of the data distribution was assessed using the Shapiro–Wilk test, which indicated that while deviation metrics in the maxillary arch were normally distributed (*p* > 0.05), some alignment protocols in the mandibular arch exhibited deviations from a normal distribution (*p* < 0.05). Consequently, descriptive statistics were reported using both mean ± standard deviation (SD) and median (interquartile range [IQR]), and minimum and maximum values.

For Phase I, inter‐scanner landmark agreement was assessed using 3D Euclidean deviations, and paired comparisons between scanners were evaluated using the Wilcoxon signed‐rank test. Intra‐observer repeatability of landmark placement was quantified using intraclass correlation coefficients (ICCs) based on a two‐way mixed‐effects model with absolute agreement.

For Phase II, the effects of the alignment protocol and dental arch on deviation outcomes were assessed using mixed‐effects modeling. Because the deviation metrics exhibited different distributional properties, the specific model types were tailored accordingly. RMS and the (90–10)/2 percentile half‐range were analyzed using a linear mixed model (LMM) assuming a normal (Gaussian) distribution with an identity link function. Absolute average deviation (Abs. Avg.) was analyzed using a generalized linear mixed model (GLMM) specifying a Gamma distribution with a log link function. All models included the alignment protocol and dental arch as fixed effects, along with their two‐way interaction. A random intercept for model identity was included to account for repeated measurements within the same model across protocols, utilizing a variance components covariance structure. Variance parameters were estimated using restricted maximum likelihood (REML) for the LMMs and residual pseudo‐likelihood (REPL) for the GLMM. Pairwise comparisons were conducted using estimated marginal means with sequential Bonferroni adjustment for multiple testing. Agreement between repeated measurements (T0 and T1) was assessed using ICC (A,1) with 95% bootstrap confidence intervals (20,000 resamples) for RMS, Abs. Avg., and (90–10)/2. Statistical analyses were performed using IBM SPSS Statistics for Windows, version 26.0 (IBM, Armonk, NY, USA). The level of significance was set at *p* < 0.05.

## 3 Results

Across the five models, both scanners demonstrated high repeatability, with a mean RMS precision value of 11 ± 2 μm for the EOS and 44 ± 3 μm for the IOS. In the pilot subset, all three predefined landmarks demonstrated excellent intra‐observer repeatability (ICC > 0.900). After rigid pose normalization (ICP), post‐registration Δ3D values remained small across landmarks (Table 1). Consistent with this methodological aim, paired axis‐wise coordinate differences between datasets were not statistically significant (Wilcoxon signed‐rank test; *p* > 0.05 for all axes).

**TABLE 1:** Landmark repeatability and coordinate consistency.

Landmark	ICC	Mean Δ3D ± SD (mm)	95% CI (mean Δ3D)	Wilcoxon *p* (Δ*x*)	Wilcoxon *p* (Δ*y*)	Wilcoxon *p* (Δ*z*)
Right canine	0.948	0.039 ± 0.018	0.027–0.052	0.770	0.432	0.275
Right central	0.911	0.035 ± 0.031	0.013–0.057	0.625	0.375	0.432
Left canine	0.976	0.030 ± 0.026	0.012–0.049	0.557	0.770	1.000

Abbreviations: CI, confidence interval; ICC, intraclass correlation coefficient; SD, standard deviation.

The descriptive statistics for the deviation metrics (RMS, Abs. Avg., and (90–10)/2) across the different alignment protocols and dental arches are summarized in Table 2. A consistent pattern was observed across all trueness metrics, where the AGB and both 3PL protocols demonstrated comparable performance, whereas the ASA protocol consistently exhibited higher deviation. In the maxilla, the AGB, 3PL_C, and 3PL_U0–U6 protocols showed similar RMS values (44 ± 8 μm, 44 ± 8 μm, and 49 ± 10 μm), whereas the ASA protocol exhibited higher deviation (58 ± 12 μm). A similar pattern was observed in the mandible, where the AGB, 3PL_C, and 3PL_U0–U6 protocols produced comparable RMS values (72 ± 44 μm, 73 ± 45 μm, and 75 ± 45 μm, respectively), whereas the ASA protocol yielded the highest mean RMS (100 ± 53 μm). The same pattern was observed in the Abs. Avg. and (90–10)/2 metrics for both arches. An evaluation of the extreme deviation ranges further supported these findings. While the global and three‐point alignment methods maintained relatively constrained maximum deviations, the ASA protocol produced the highest maximum errors. This was most pronounced in the mandibular arch, where the maximum observed RMS for ASA reached 190 μm, compared to a maximum of 160 μm for the AGB, 3PL_C, and 3PL_U0–U6 protocols.

**TABLE 2:** Descriptive statistics for deviation metrics by arch and alignment protocol.

Alignment protocol	RMS (μm)			Abs. Avg. (μm)			(90–10)/2 (μm)			Maximum positive deviation (μm)			Maximum negative deviation (μm)		
	Mean ± SD	Median (IQR)	Min; max	Mean ± SD	Median (IQR)	Min; max	Mean ± SD	Median (IQR)	Min; max	Mean ± SD	Median (IQR)	Min; max	Mean ± SD	Median (IQR)	Min; Max
Maxilla															
AGB	44 ± 8	45 (10)	30; 50	36 ± 6	36 (10)	30; 50	58 ± 11	57 (20)	40; 70	118 ± 48	106 (20)	70; 250	−119 ± 48	−109 (10)	−250; −70
3PL _C	44 ± 8	45 (10)	30; 50	35 ± 5	36 (10)	30; 40	56 ± 10	57 (20)	40; 70	123 ± 48	107 (40)	70; 250	−117 ± 49	−104 (20)	−250; −70
3PL_U0–U6	49 ± 10	52 (20)	30; 60	38 ± 8	38 (10)	30; 50	62 ± 14	63 (20)	40; 80	123 ± 48	107 (40)	70; 250	−117 ± 49	−104 (20)	−250; −70
ASA	58 ± 12	57 (20)	40; 80	44 ± 8	45 (10)	30; 60	73 ± 15	73 (20)	50; 100	147 ± 56	133 (60)	100; 270	−154 ± 72	−139 (90)	−290; −70
Mandible															
AGB	72 ± 44	53 (80)	30; 160	56 ± 32	43 (60)	30; 120	88 ± 49	69 (90)	40; 180	192 ± 147	147 (160)	80; 570	−254 ± 213	−176 (240)	−740; −70
3PL_C	73 ± 45	52 (90)	30; 160	57 ± 34	43 (70)	30; 120	89 ± 51	69 (100)	40; 180	192 ± 149	142 (150)	90; 580	−185 ± 129	−143 (150)	−490; −70
3PL_U0–U6	75 ± 45	54 (80)	30; 160	58 ± 33	43 (60)	30; 120	90 ± 50	69 (90)	40; 180	192 ± 149	142 (150)	90; 580	−185 ± 129	−143 (150)	−490; −70
ASA	100 ± 53	79 (100)	50; 190	72 ± 36	59 (70)	40; 140	114 ± 57	91 (100)	60; 220	292 ± 162	234 (230)	140; 620	−195 ± 141	−147 (140)	−550; −70

Abbreviations: 3PL, three‐point landmark alignment; AGB, automatic global best‐fit alignment; ASA, area‐specific alignment; IQR, interquartile range; Max, maximum; Min, minimum; SD, standard deviation.

**FIGURE 2:**
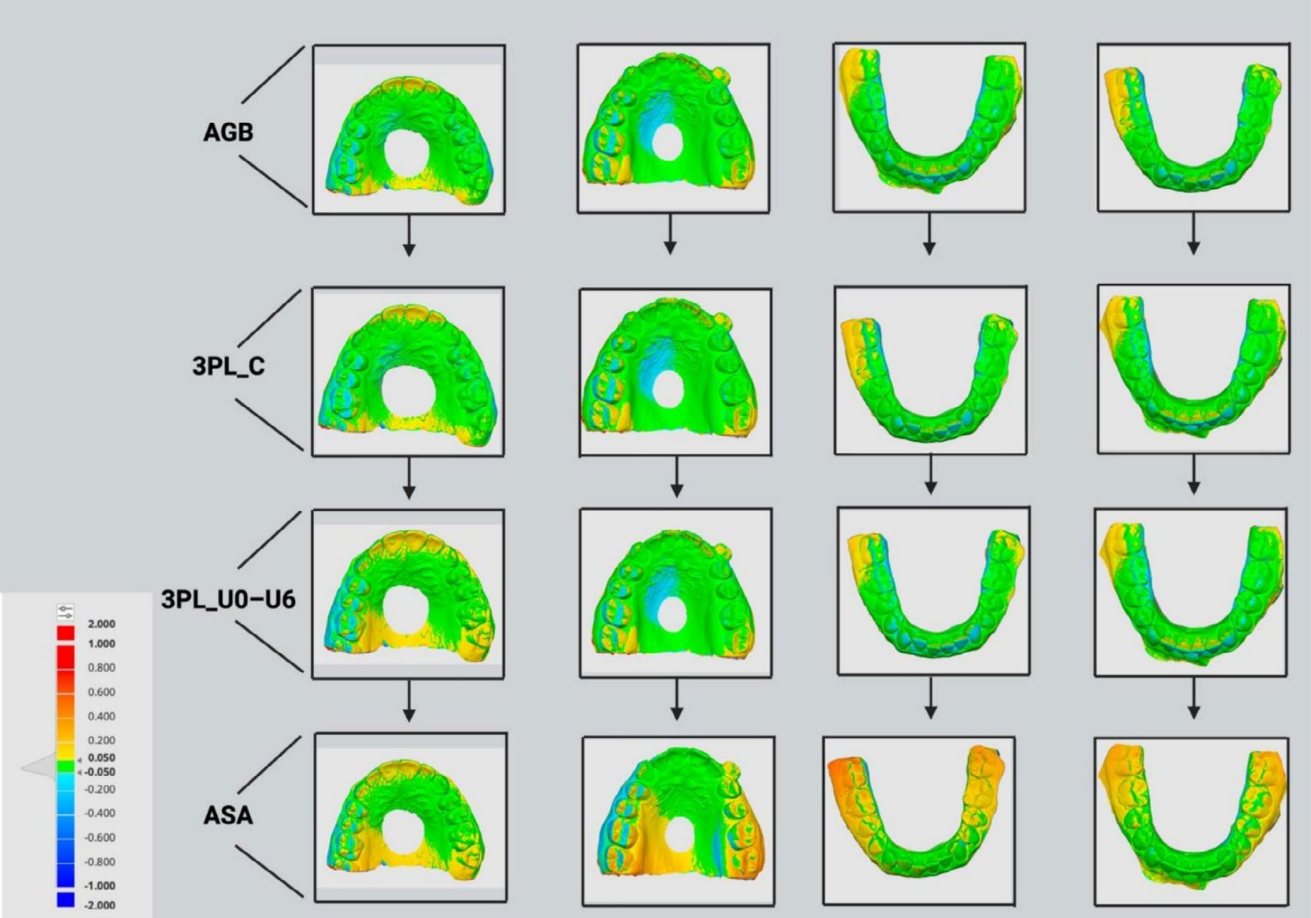
Representative color‐coded deviation maps of maxillary and mandibular full‐arch models across superimposition protocols.

The mixed‐effects model analyses revealed a significant main effect of the alignment protocol on all trueness metrics (*p* < 0.001). The main effect of the dental arch was significant for the RMS metric (*p* = 0.045), with the mandible exhibiting higher overall deviation than the maxilla. Furthermore, a significant interaction between the alignment protocol and the dental arch was observed for both the RMS (*p* < 0.001) and (90–10)/2 (*p* = 0.046) metrics, whereas no significant interaction was found for the Abs. Avg. metric (*p* = 0.484) (Table 3). Pairwise comparisons demonstrated that the AGB, 3PL_C, and 3PL_U0–U6 alignment protocols yielded comparable trueness, with no differences found among them in either the maxilla or the mandible across any deviation metric (*p* > 0.05). This indicates that using a wider molar‐guided reference triangle (3PL_U0–U6) did not significantly improve trueness compared to the narrower anterior canine‐guided triad (3PL_C). However, the ASA alignment protocol resulted in lower trueness than the other methods (*p* < 0.01), with the magnitude of the error introduced by the ASA protocol varying by arch. In the maxilla, the ASA protocol resulted in a mean RMS increase of 13 μm compared to the AGB protocol (*p* < 0.001), whereas in the mandible, this difference was much more pronounced, with ASA increasing the RMS error by 27 μm compared to the AGB protocol (*p* < 0.001). Similar differences were also observed for the Abs. Avg. and (90–10)/2 metrics, confirming that the ASA alignment protocol consistently resulted in lower trueness compared to the global and three‐point strategies (Table 4). Repeatability was excellent across protocols (ICC (A,1) = 0.971–0.997) (Table 5). Figure 2 illustrates this pattern, with broadly comparable surface agreement for the AGB and both three‐point landmark protocols, and more extensive, localized deviations for the ASA protocol, particularly in the mandible.

**TABLE 3:** Results of the mixed‐effects models.

	Num df	Den df	RMS^a^		Abs. Avg.^b^		(90–10)/2^a^	
			*F*	*p*	*F*	*p*	*F*	*p*
Alignment protocol	3	72	52.724	< 0.001***	36.847	< 0.001***	34.570	< 0.001***
Arch	1	72	4.161	0.045*	3.410	0.069	3.839	0.054
Alignment protocol × Arch	3	72	7.472	< 0.001***	0.826	0.484	2.806	0.046*

Abbreviations: Den df, denominator degrees of freedom; *F*, *F* statistic; Num df, numerator degrees of freedom.

^a^ Analyzed using a linear mixed model (LMM).

^b^ Analyzed using a generalized linear mixed model (GLMM).

* *p* < 0.05.

*** *p* < 0.001.

**TABLE 4:** Pairwise comparisons of alignment protocols.

Comparison	RMS (μm)^a^			Abs. Avg. (μm)^b^			(90–10)/2 (μm)^a^		
	MD ± SE	*p*	95% CI	MD ± SE	*p*	95% CI	MD ± SE	*p*	95% CI
			Lower; upper			Lower; upper			Lower; upper
Maxilla									
AGB vs. 3PL_C	< 1 ± 3	0.881	−5; 6	1 ± 1	0.673	−2; 3	1 ± 3	0.723	−6; 8
AGB vs. 3PL_U0–U6	−5 ± 3	0.179	−11; 1	−2 ± 2	0.313	−6; 1	−4 ± 3	0.479	−12; 4
AGB vs. ASA	−13 ± 3	< 0.001^c^	−20; −6	−9 ± 2	< 0.001^c^	−14; −4	−15 ± 3	< 0.001^c^	−24; −6
3PL_C vs. 3PL_U0–U6	−5 ± 3	0.179	−12; 1	−3 ± 2	0.215	−7; 1	−5 ± 3	0.383	−13; 3
3PL_C vs. ASA	−14 ± 3	< 0.001^c^	−21; −6	−10 ± 2	< 0.001^c^	−15; −4	−16 ± 3	< 0.001^c^	−26; −7
3PL_U0–U6 vs. ASA	−9 ± 3	0.008^c^	−15; 2	−7 ± 2	0.003^c^	−12; −2	−11 ± 3	0.006^c^	−20; −3
Mandible									
AGB vs. 3PL_C	−1 ± 3	0.892	−6; 5	< 1 ± 2	0.817	−4; 4	−2 ± 3	1.000	−9; 6
AGB vs. 3PL_U0–U6	−3 ± 3	0.892	−9; 4	−2 ± 2	0.796	−7; 3	−2 ± 3	1.000	−11; 6
AGB vs. ASA	−27 ± 3	< 0.001^c^	−35; −20	−17 ± 3	< 0.001^c^	−25; −8	−26 ± 3	< 0.001^c^	−35; −17
3PL_C vs. 3PL_U0–U6	−2 ± 3	0.892	−8; 4	−2 ± 2	0.796	−7; 3	−1 ± 3	1.000	−8; 6
3PL_C vs. ASA	−27 ± 3	< 0.001^c^	−34; −20	−16 ± 3	< 0.001^c^	−25; −8	−25 ± 3	< 0.001^c^	−34; −16
3PL_U0–U6 vs. ASA	−25 ± 3	< 0.001^c^	−31; −18	−14 ± 3	< 0.001^c^	−22; −7	−24 ± 3	< 0.001^c^	−33; −15

Abbreviations: 3PL, three‐point landmark alignment; AGB, automatic global best‐fit alignment; ASA, area‐specific alignment; CI, confidence interval; MD, mean difference; SE, standard error.

^a^ Analyzed using a linear mixed model (LMM).

^b^ Analyzed using a generalized linear mixed model (GLMM).

^c^ Sequential Bonferroni adjusted significance level at *p* < 0.05.

**TABLE 5:** Intra‐observer repeatability of superimposition protocols.

Protocol	RMS ICC (95% CI)	Abs Avg ICC (95% CI)	(90–10)/2 ICC (95% CI)
AGB	0.996 (0.976–1.000)	0.992 (0.962–1.000)	0.990 (0.955–1.000)
3PL_C	0.997 (0.987–0.999)	0.997 (0.980–1.000)	0.995 (0.964–0.999)
3PL_U0–U6	0.997 (0.985–0.999)	0.997 (0.970–1.000)	0.993 (0.961–0.999)
ASA	0.971 (0.881–0.991)	0.973 (0.870–0.989)	0.988 (0.927–0.998)

Abbreviations: CI, confidence interval; ICC, intraclass correlation coefficient.

## 4 Discussion

This study investigated how registration strategy influences surface deviation outcomes in clinically derived orthodontic full‐arch models by intentionally separating landmark validation from trueness testing. Phase I verified landmark suitability by quantifying intra‐observer repeatability and inter‐scanner landmark concordance (Δ3D). Phase II then compared reference‐based surface trueness metrics across superimposition paradigms. The key finding was that the choice of superimposition paradigm substantially altered deviation metrics even when landmark concordance between scanners was high, underscoring that superimposition is not a neutral preprocessing step but an outcome‐determining component of the digital workflow.

A recent study has indicated that registration accuracy can be sensitive to upstream processing choices and may show axis‐dependent behavior, with discrepancies often more pronounced in the apicocoronal (vertical) dimension [15]. On this basis, we performed a pilot coordinate‐based screening to identify clinically reproducible landmarks with minimal inter‐scanner displacement, reasoning that selecting the most concordant control points would strengthen the robustness of subsequent 3PL initialization and reduce alignment‐related variability. In the pilot phase, the selected landmarks showed high intra‐observer repeatability and small post‐registration 3D Euclidean discrepancies, with no axis‐wise paired coordinate differences between scanners. These findings indicate that canine cusp tips and an incisal edge point were consistently identifiable across datasets and sufficiently stable to function as control points for a landmark‐guided workflow. Accordingly, the small Δ3D values and the absence of systematic axis‐wise differences should be interpreted as evidence that landmark placement did not introduce a directional bias likely to confound subsequent protocol comparisons. This validation strengthens the interpretation of Phase II by supporting that any observed differences in surface‐based trueness metrics are more plausibly attributable to registration paradigm behavior than to instability or inconsistency in landmark definition. It should also be noted that these favorable Phase I results were obtained after ICP‐based pose normalization, which likely improved comparability of coordinates across datasets. Thus, the observed repeatability reflects not only landmark reproducibility, but also the stabilizing effect of prior rigid registration.

A major source of methodological heterogeneity in the literature is that 3PL alignment is frequently used primarily for initialization, after which an automated surface‐based refinement is applied [6, 7, 9, 14]. As a result, it can be difficult to attribute reported deviation outcomes to the user‐defined constraint rather than to the optimizer's convergence behavior. This distinction is particularly relevant because systematic evidence has highlighted a persisting methodological gap and the lack of consensus on universally reliable dental landmarks [4, 12, 13]. Within this context, hybrid approaches remain compelling in practice, but their interpretability depends on transparent reporting and on using landmarks that are demonstrably repeatable. In the present study, 3PL was implemented as landmark‐based initialization followed by the software's automated ICP refinement. Accordingly, 3PL should be interpreted as a hybrid workflow rather than a purely landmark‐only rigid transformation. To reduce operator‐related variability within this hybrid framework, the candidate landmarks in Phase I were first validated and then implemented in the 3PL using both a pilot‐validated triad and the commonly used U0–U6 occlusal reference triangle as a sensitivity check of landmark‐set selection. Accordingly, the term “landmark distribution” in the present study should be interpreted as referring to these alternative three‐point landmark configurations rather than to a broader standalone landmark‐based registration paradigm.

In the main analysis, deviation outcomes were influenced by the alignment protocol, which demonstrated a significant main effect across all trueness metrics. Furthermore, a significant protocol‐by‐arch interaction was observed for the RMS and (90–10)/2 metrics (though not for Abs. Avg.), indicating that the magnitude of protocol‐related error was highly arch‐dependent, with the mandible exhibiting higher deviations when ASA was applied. In contrast to our findings, a typodont study showed that area‐based best‐fit achieved the highest trueness and precision, while landmark‐based methods performed worse [8]. However, these results were derived from a typodont model that used area‐based alignment on the surface of 3 denture teeth and was limited to the mandible. Therefore, its direct comparability to our patient‐derived full‐arch results may be limited. More broadly, prior dental studies consistently indicate that superimposition outcomes are sensitive to the chosen alignment algorithm, yet direct cross‐study comparisons are often constrained by methodological heterogeneity (e.g., differences in scan geometry and extent, the number and definition of reference points/areas, and the primary objective, such as wear quantification versus scanner accuracy) [6, 8, 9, 16, 18, 19].

A consistent pattern emerged in this study, demonstrating that AGB and 3PL produced comparable outcomes, whereas anatomically restricted ASA consistently resulted in higher deviations. The similarity between AGB and 3PL should be interpreted in light of their shared reliance on surface‐based optimization. In our implementation, 3PL and ASA differed from AGB primarily in the initial pose initialization, while both workflows ultimately relied on automated surface refinement. Under full‐arch conditions with extensive shared geometry and consistent landmark identification, the comparable deviation metrics observed for these workflows may reflect the influence of the subsequent surface‐based refinement step. Importantly, this does not imply that landmark constraints alone replicate global best‐fit behavior; rather, the observed similarity may reflect the combined effect of landmark initialization and subsequent automated surface refinement under full‐arch conditions. ASA restricts initial optimization to a limited anatomical region, which can overweight local surface characteristics and shift mismatch to non‐referenced portions of the arch, thereby inflating whole‐arch deviation metrics. This effect was numerically larger in the mandible, consistent with the absence of a universally accepted stable mandibular reference region analogous to palatal rugae in the maxilla [20, 21, 22]. Consequently, restricted‐area registration may be more vulnerable to region‐definition choices, local surface variability, and boundary effects in mandibular full‐arch datasets. Collectively, these results indicate that reduced reference regions were associated with higher whole‐arch deviations in the present dataset, despite high landmark repeatability. To better contextualize the present findings within the existing literature, a summary comparison of relevant previous studies is provided in Table 6.

**TABLE 6:** Comparison of the present study with relevant previous studies on digital dental model superimposition.

Study	Model/study design	Registration strategy	Main outcome(s)	Key finding(s)	Relation to the present study
Present study	In vitro full‐arch orthodontic models; maxilla and mandible; intraoral scanner vs. desktop scanner	Automatic global best‐fit, three‐point landmark‐based initialization with refinement, and area‐specific alignment	RMS, absolute average deviation, (90–10)/2, min, max	Automatic global best‐fit and both three‐point protocols showed comparable trueness; area‐specific alignment produced significantly greater deviations, especially in the mandible	Demonstrated that the superimposition protocol itself influenced full‐arch deviation outcomes
Revilla‐León et al., 2023	In vitro typodont mandibular model	Entire‐dataset best‐fit, section‐based best‐fit, and landmark‐based methods	RMS	The best‐fit algorithms influenced alignment discrepancy; entire‐dataset and section‐based best‐fit methods showed higher deviations than landmark‐based alignment	Supports the importance of registration strategy, although the model type, anatomical scope, and workflow differed from the present study
O'Toole et al., 2019	In vitro 3D dental scans of 10 natural molar teeth	Best‐fit, landmark‐based, and reference alignment methods	Translation error and angular error (original vs. digitally eroded surface)	Alignment procedures affected both alignment accuracy and downstream measurement outcomes	Supports the present conclusion that superimposition is not a neutral preprocessing step and can alter results
Negm et al., 2024	In vitro Partially dentate maxilla model	Automatic alignment of scans, followed by subsequent local best‐fit using the 3 different reference areas	RMS deviations	The selected reference area affected measured scanning accuracy	Consistent with the present finding that restricted reference can influence deviation outcomes
Loumprinis et al., 2024	In vitro semi‐arch erosive tooth wear models	Best‐fit, three‐point alignment, and palatal rugae	Surface profile differences	Different alignment methods produced method‐dependent measurement behavior, without one method being uniformly superior across all conditions	Reinforces that measured outcomes depend on alignment strategy, recommends choosing the most suitable scan alignment cases basis
Vargas‐Quiroga et al., 2025	Clinical mandibular digital models	5 Different alignment methods	RMS	Alignment strategy influenced mandibular model trueness	Supports the present observation that mandibular outcomes are especially sensitive to registration strategy
Becker et al., 2018	In vitro maxillary dental models	Landmark‐based registration, ICP	RMS errors and Hausdorff	Registration reliability depended on accurate manual control point selection; ICP refinement improved matching	Supports the interpretation that reliable superimposition of digital dental Models depends on strategy

Notably, these findings should be interpreted in the context of how “reference alignment” is used in other domains. It has been shown that restricting best‐fit to presumed unchanged surfaces can improve measurement trueness when a stable, sufficiently informative reference region exists. However, in cross‐scanner full‐arch comparisons, restricting alignment to a small or variably captured region may instead increase sensitivity to local noise and differences in surface representation, thereby elevating global deviation outcomes [10]. Thus, the performance of restricted‐area approaches is likely condition‐dependent and strongly influenced by the stability and distinctiveness of the chosen reference region. When clinical change between scans is expected, the choice of registration strategy may require additional consideration, because different alignment workflows may influence how measured differences are represented. As the literature has also suggested, deviation values tend to increase with the scanned surface area, particularly when moving from localized unilateral reference regions to arch‐level scans, making it challenging to identify a consistently stable and universally reliable reference area. For this reason, until a truly dependable anatomical reference region is established, workflows based on global best‐fit and/or three‐point guidance may remain more appropriate for applications that demand highly sensitive measurements. In practice, clinicians can choose among these three approaches based on the level of precision required. Although ASA differed statistically from the other methods in our study, the magnitude of this difference was approximately 20 μm, which may or may not be clinically meaningful, depending on the expected sensitivity and the clinician's tolerance threshold.

In alignment with established protocols that identify extraoral desktop scanners as reliable reference standards [8, 26], the DuraScan Alpha was used to generate the reference datasets. To ensure the scanner's suitability for trueness estimation, internal precision was verified by repeated scans to assess reference repeatability. In addition, repeated‐scan precision assessments indicated that both TRIOS and DuraScan demonstrated high repeatability under the present experimental conditions. This strengthens confidence that the observed Phase II differences primarily reflect registration paradigm behavior rather than acquisition instability.

These findings have implications for both research standardization and clinical digital workflows. Reported trueness outcomes are not directly comparable across studies unless alignment strategies are explicitly defined and methodologically aligned. In clinically derived full‐arch orthodontic datasets, different paradigms can produce meaningfully different deviation metrics, and restricted‐area approaches may increase deviations if the chosen region is not sufficiently stable or distinctive, particularly in the mandible. Clinically, this matters for longitudinal record management, archival cast digitization, and cross‐platform comparisons, where alignment choices can influence numeric interpretations derived from the same underlying anatomy.

This study has several limitations that warrant consideration. The in vitro design of this study does not allow for the reproduction of various intraoral conditions (saliva, soft tissue dynamics, patient movement, and optical artifacts), which may further influence scan quality and alignment behavior clinically. The sample size, while typical for controlled full‐arch analyses, may not capture the full spectrum of orthodontic morphological variability. Results are also software‐ and workflow‐dependent. Importantly, because 3PL was followed by automated ICP refinement, the present design cannot fully disentangle the relative contributions of landmark initialization from those of surface‐based convergence. Future studies should replicate analyses across software platforms, test additional mandibular reference regions and boundary definitions for ASA, and incorporate designs that explicitly isolate the contributions of initialization versus refinement where feasible.

## 5 Conclusion

Within the limitations of this study, the candidate dental landmarks demonstrated high repeatability and were suitable for landmark‐guided initialization. The first hypothesis was supported. Full‐arch deviation outcomes varied according to the superimposition protocol. AGB and three‐point landmark registration showed comparable trueness values in both arches, whereas the area‐specific protocol showed significantly greater deviations. The second hypothesis was partially supported. The effect of the superimposition protocol differed between the maxillary and mandibular arches. The deviation increase observed with the area‐specific protocol was greater in the mandible than in the maxilla.

## Funding

The authors have nothing to report.

## Conflicts of Interest

The authors declare no conflicts of interest.

## Data Availability

The data that support the findings of this study are available from the corresponding author upon reasonable request.

## References

[1] V. D'Antò , R. Rongo , S. D. Casaburo , et al., “Predictability of Tooth Rotations in Patients Treated With Clear Aligners,” Scientific Reports 14, no. 1 (2024): 11348, 10.1038/s41598-024-61594-2. 38762583 PMC11102536

[2] J. Li , T. Joda , M. Revilla‐León , M. H. A. Saleh , Z. Chen , and H. Wang , “Recommendations for Successful Virtual Patient‐Assisted Esthetic Implant Rehabilitation: A Guide for Optimal Function and Clinical Efficiency,” Journal of Esthetic and Restorative Dentistry 36, no. 1 (2024): 186–196, 10.1111/jerd.13142. 37792734

[3] S. Gracis , A. Appiani , and G. Noè , “Digital Workflow in Implant Prosthodontics: The Critical Aspects for Reliable Accuracy,” Journal of Esthetic and Restorative Dentistry 35, no. 1 (2023): 250–261, 10.1111/jerd.13004. 36606714

[4] S. Stucki and N. Gkantidis , “Assessment of Techniques Used for Superimposition of Maxillary and Mandibular 3D Surface Models to Evaluate Tooth Movement: A Systematic Review,” European Journal of Orthodontics 42, no. 5 (2020): 559–570, 10.1093/ejo/cjz075. 31742598

[5] International Organization for Standardization , Accuracy (Trueness and Precision) of Measurement Methods and Results—Part 1: General Principles and Definitions (International Organization for Standardization, 2023), Report.

[6] E. E. Negm , M. Patel , and P. Ryan , “Impact of the Superimposition Reference Area on Intraoral Scanning Accuracy in a Partially Dentate Maxilla,” Journal of Prosthetic Dentistry 132, no. 1 (2024): 189.e1–189.e11, 10.1016/j.prosdent.2024.03.018. 38556406

[7] S. Nada , M. Hasanin , and R. ElNaghy , “A Critical Evaluation of Image Superimposition in Dentistry,” Journal of Dental Research 104, no. 5 (2025): 465–472, 10.1177/00220345241311263. 39953709

[8] M. Revilla‐León , A. Gohil , A. B. Barmak , A. Zandinejad , A. J. Raigrodski , and P.‐B. J. Alonso , “Best‐Fit Algorithm Influences on Virtual Casts' Alignment Discrepancies,” Journal of Prosthodontics 32, no. 4 (2023): 331–339, 10.1111/jopr.13537. 35524587

[9] S. O'Toole , C. Osnes , D. Bartlett , and A. Keeling , “Investigation Into the Accuracy and Measurement Methods of Sequential 3D Dental Scan Alignment,” Dental Materials 35, no. 3 (2019): 495–500, 10.1016/j.dental.2019.01.012. 30683418

[10] N. Loumprinis , S. Michou , and C. Rahiotis , “Different Methods of Scan Alignment in Erosive Tooth Wear Measurements: An In Vitro Study,” Dentistry Journal (Basel) 12, no. 2 (2024): 34, 10.3390/dj12020034. PMC10887586 38392238

[11] W. G. Renne , Z. P. Evans , A. Mennito , and M. Ludlow , “A Novel Technique for Reference Point Generation to Aid in Intraoral Scan Alignment,” Journal of Esthetic and Restorative Dentistry 29, no. 6 (2017): 391–395, 10.1111/jerd.12316. 28653808

[12] F. Kernen , S. Schlager , V. Seidel Alvarez , et al., “Accuracy of Intraoral Scans: An In Vivo Study of Different Scanning Devices,” Journal of Prosthetic Dentistry 128, no. 6 (2022): 1303–1309, 10.1016/j.prosdent.2021.03.007. 33902891

[13] M. A. Akl , K. Daifallah , J. A. Pérez‐Barquero , A. B. Barmak , A. G. Wee , and M. Revilla‐León , “Influence of Interdental Spaces and the Palate on the Accuracy of Maxillary Scans Acquired Using Different Intraoral Scanners,” Journal of Prosthodontics 32, no. S2 (2023): 125–134, 10.1111/jopr.13748. 37591814

[14] F. Zarone , G. Ruggiero , M. Ferrari , F. Mangano , T. Joda , and R. Sorrentino , “Accuracy of a Chairside Intraoral Scanner Compared With a Laboratory Scanner for the Completely Edentulous Maxilla: An In Vitro 3‐Dimensional Comparative Analysis,” Journal of Prosthetic Dentistry 124, no. 6 (2020): 761.e1–761.e7, 10.1016/j.prosdent.2020.07.018. 33289647

[15] L. Lo Russo , M. Lorusso , C. Ercoli , et al., “Effect of the CBCT Data Segmentation Threshold on Registration Accuracy With Surface Scanning,” Journal of Prosthetic Dentistry 135 (2025): 595.e1–595.e10, 10.1016/j.prosdent.2025.10.044. 41198479

[16] M. Revilla‐León , J. A. Pérez‐Barquero , B. A. Barmak , R. Agustín‐Panadero , L. Fernández‐Estevan , and W. Att , “Facial Scanning Accuracy Depending on the Alignment Algorithm and Digitized Surface Area Location: An In Vitro Study,” Journal of Dentistry 110 (2021): 103680, 10.1016/j.jdent.2021.103680. 33901605

[17] M. Revilla‐León , R. Aragoneses , E. M. Arroyo Valverde , M. Gómez‐Polo , and J. C. Kois , “Classification of Scanning Errors of Digital Scans Recorded by Using Intraoral Scanners,” Journal of Esthetic and Restorative Dentistry 37, no. 6 (2025): 1363–1371, 10.1111/jerd.13419. 39828965

[18] E. D. Vargas‐Quiroga , M. B. Lemos Reis , J. J. Faraoni , A. B. Novaes , R. G. Palma‐Dibb , and B. S. H. Tonin , “Alignment Methods to Improve the Trueness of Digital Mandibular Models: A Clinical Study,” Digital Dentistry Journal 2, no. 2 (2025): 100046, 10.1016/j.ddj.2025.100046.

[19] K. Becker , B. Wilmes , C. Grandjean , and D. Drescher , “Impact of Manual Control Point Selection Accuracy on Automated Surface Matching of Digital Dental Models,” Clinical Oral Investigations 22, no. 2 (2018): 801–810, 10.1007/s00784-017-2155-6. 28681247

[20] T. Castroflorio , S. Avolese , F. Sanna , and S. Parrini , “The Role of Stable Anatomical Landmarks in Automated 3D Model Superimposition: A Closer Look,” Bioengineering (Basel) 12, no. 8 (2025): 839, 10.3390/bioengineering12080839. 40868352 PMC12383528

[21] S. M. Adel , N. R. Vaid , N. El‐Harouni , H. Kassem , and A. R. Zaher , “Digital Model Superimpositions: Are Different Software Algorithms Equally Accurate in Quantifying Linear Tooth Movements?,” BMC Oral Health 22, no. 1 (2022): 103, 10.1186/s12903-022-02129-x. 35361187 PMC8973572

[22] J. I. Choi , B. K. Cha , P. G. Jost‐Brinkmann , D. S. Choi , and I. S. Jang , “Validity of Palatal Superimposition of 3‐Dimensional Digital Models in Cases Treated With Rapid Maxillary Expansion and Maxillary Protraction Headgear,” Korean Journal of Orthodontics 42, no. 5 (2012): 235–241, 10.4041/kjod.2012.42.5.235. 23173116 PMC3495254

[23] G. Fiorillo , A. Campobasso , G. Caldara , et al., “Accuracy of 3‐Dimensional–Printed Customized Transfer Tray Using a Flash‐Free Adhesive System in Digital Indirect Bonding: An In Vivo Study,” American Journal of Orthodontics and Dentofacial Orthopedics 164, no. 4 (2023): 505–515, 10.1016/j.ajodo.2023.02.017. 37074245

[24] Y. G. Moon and K. M. Lee , “Comparison of the Accuracy of Intraoral Scans Between Complete‐Arch Scan and Quadrant Scan,” Progress in Orthodontics 21, no. 1 (2020): 36, 10.1186/s40510-020-00337-1. 33000308 PMC7527390

[25] J. Li , P. Yuan , C. M. Chang , et al., “New Approach to Establish an Object Reference Frame for Dental Arch in Computer‐Aided Surgical Simulation,” International Journal of Oral and Maxillofacial Surgery 46, no. 9 (2017): 1193–1200, 10.1016/j.ijom.2017.04.012. 28499508 PMC5559304

[26] M. G. Kanmaz , G. Agani Sabah , M. Balcı , and M. B. Erden , “Comparison of Intraoral Scanner Accuracy Before and After Calibration: An In Vitro Study,” BMC Oral Health 25, no. 1 (2025): 1186, 10.1186/s12903-025-06584-0. 40676523 PMC12273024

